# Psychobiological indicators of the subjectively experienced health status - findings from the Women 40+ Healthy Aging Study

**DOI:** 10.1186/s12905-020-0888-x

**Published:** 2020-01-29

**Authors:** Serena Fiacco, Laura Mernone, Ulrike Ehlert

**Affiliations:** 1 Clinical Psychology and Psychotherapy, University of Zurich, Binzmühlestrasse 14, 8050 Zurich, Switzerland; 2 URPP Dynamics of Healthy Aging Research Priority Program, University of Zurich, Zurich, Switzerland

**Keywords:** Healthy aging, women’s health, BMI, Physical activity, Steroid hormones

## Abstract

**Background:**

Healthy aging is particularly important in women, as their life-span is generally longer than men’s, leaving women at higher risk for age-related diseases. Understanding determinants of women’s healthy aging is therefore a major public health interest. Clinical utility of previous research is limited, through its focus on either single psychosocial or biological predictors. The present study investigated psychobiological predictors of women’s healthy aging, for the first time including positive psychological traits and biomarkers of healthy aging.

**Methods:**

Totally, 121 generally healthy women aged 40 to 75 were investigated cross-sectionally. Healthy aging was operationalized via self-rated health (SRH). To gain a nuanced view of the particularities at the upper end of the illness-wellness continuum, women with excellent SRH and those with good SRH were analyzed as distinct groups. Socioeconomic and sociodemographic variables, health behavior, resilience, optimism, and self-worth as well as menopausal symptoms, and levels of steroid hormones and gonadotropins were considered as predictors of SRH. Binary logistic regression analyses using the forward conditional method were performed with the two health status groups as dependent variable.

**Results:**

Women with a lower body mass index (BMI; OR = .59, 95% CI = .33–1.03), higher intensive physical activity (OR = 2.27, 95% CI = 1.06–4.86), and higher resilience (OR = 2.37, 95% CI = 1.34–4.18) were more likely to rate their health as excellent compared to good. No clinically significant differences could be found regarding endocrine levels.

**Conclusion:**

Psychobiological indicators (lower BMI, intensive physical activity, higher resilience) discriminated SRH at the top level of the health spectrum. In healthy women, the predictive value of endocrine markers seems to be secondary. Interventions targeting these indicators could promote women’s healthy aging.

## Background

In 2017, one in five people in Europe and North America were aged 60 and above. By 2030, older individuals are expected to outnumber children younger than 10. All over the world, people are reaching older ages than previous generations [1], leading the WHO to pronounce a Decade of Action on Healthy Aging from 2020 to 2030 [2].

The notion of healthy aging goes beyond the avoidance of disability and disease. Health is a dynamic concept, which incorporates the biological, psychological, and social perspective [3]. *Normal aging* is associated with a decline in physical, social, and cognitive function [4]. Contrary to this, *healthy aging* is characterized as involving a low risk of disease and disability, high cognitive and physical functioning, and an active engagement in life [4, 5]. As women have a longer life expectancy than men, they will make up a large proportion of the older society [6, 7]. Moreover, as they reach older ages than men, women are also more prone to develop debilitating diseases [6]. Understanding determinants of women’s healthy aging is therefore a major public health interest.

A suitable way to operationalize healthy aging is by means of self-rated health, because it represents an inclusive and holistic measure incorporating components from a biologically, socially, and culturally influenced context [8]. Questions on self-rated health are simple to apply [8] and subjective health measures proofed to have high predictive power for future health [8–12]. When people are asked to indicate their self-rated health, they decide for themselves which factors to consider [13, 14], making it an inclusive and personalized approach to operationalize healthy aging.

To understand the mechanisms underlying a prolonged health span in midlife women, knowledge about the predictors of healthy aging is essential. One objective of previous investigations was therefore to find variables which can predict a high level of functioning and wellbeing in midlife and older age [15]. Studies have investigated diverse psychological or physiological variables, or combinations thereof, in order to predict healthy aging. Unfortunately, little consensus has been reached. So far, demographic characteristics and health behavior have received the most research attention. Marriage and a higher socioeconomic status have often been considered as predictors, but have yielded inconsistent associations with healthy aging (reviewed in [16]). Several studies found that a normal body mass index (BMI) and regular physical activity were promising predictors of current health [17] and future health [18, 19]. Additionally, longitudinal and cross-sectional studies revealed a positive effect of lower blood pressure and greater grip strength on healthy aging (reviewed in [16]).

Throughout their life span, women are confronted with much stronger fluctuations in hormones of the hypothalamic-pituitary-gonadal (HPG) axis than men. These fluctuations can lead to bothersome psychological and somatic symptoms and are thought to contribute to pathologies such as a major depression in vulnerable women [20–22]. Menopause is a key transitional phase in midlife, and the years preceding menopause are accompanied by even more drastic fluctuations in steroid hormones and gonadotropins than in younger years [23]. These hormonal fluctuations in midlife have been linked to psychological symptoms (i.e. anxiety and depression) as well as physiological symptoms (i.e. vasomotor symptoms and vaginal dryness) [24–27]. The endocrine contributions to positive health in midlife and older women are, however, relatively unknown. During the menopausal transition, depressive symptoms have been linked to estradiol fluctuations [28] and lower testosterone levels [29]. Moreover, higher or more stable than average estradiol levels in midlife women have been associated with higher cognitive function [30, 31]. Therefore, from an endocrinological perspective, specific profiles of steroid hormones and gonadotropins might be more favorable for women’s healthy aging than others (for review [32]).

On a psychological level, positive traits such as optimism, resilience, and self-esteem seem to mediate the association between life challenges and the maintenance of health and wellbeing [33, 34]. Optimistic people generally have positive expectations about life [35] and seem to recover faster from surgeries or acute disease (reviewed in [36]). Resilient individuals manage to keep a stable equilibrium in the face of loss or trauma [34], and high resilience was previously associated with high subjectively rated healthy aging [15]. Indeed, in the study by Jeste et al. [15], resilience explained a comparable amount of variance in successful healthy aging to subjective physical health. Self-esteem describes a person’s self-evaluation or self-appraisal [37], and longitudinal studies suggest that it is not only a consequence but also a cause of positive life outcomes [38]. Self-esteem seems to influence reports of physical health [39, 40], mental health [41], and subjective wellbeing [42]. Positive psychological traits may therefore also be predictive of healthy aging.

### Summary and research question

**Fig. 1:**
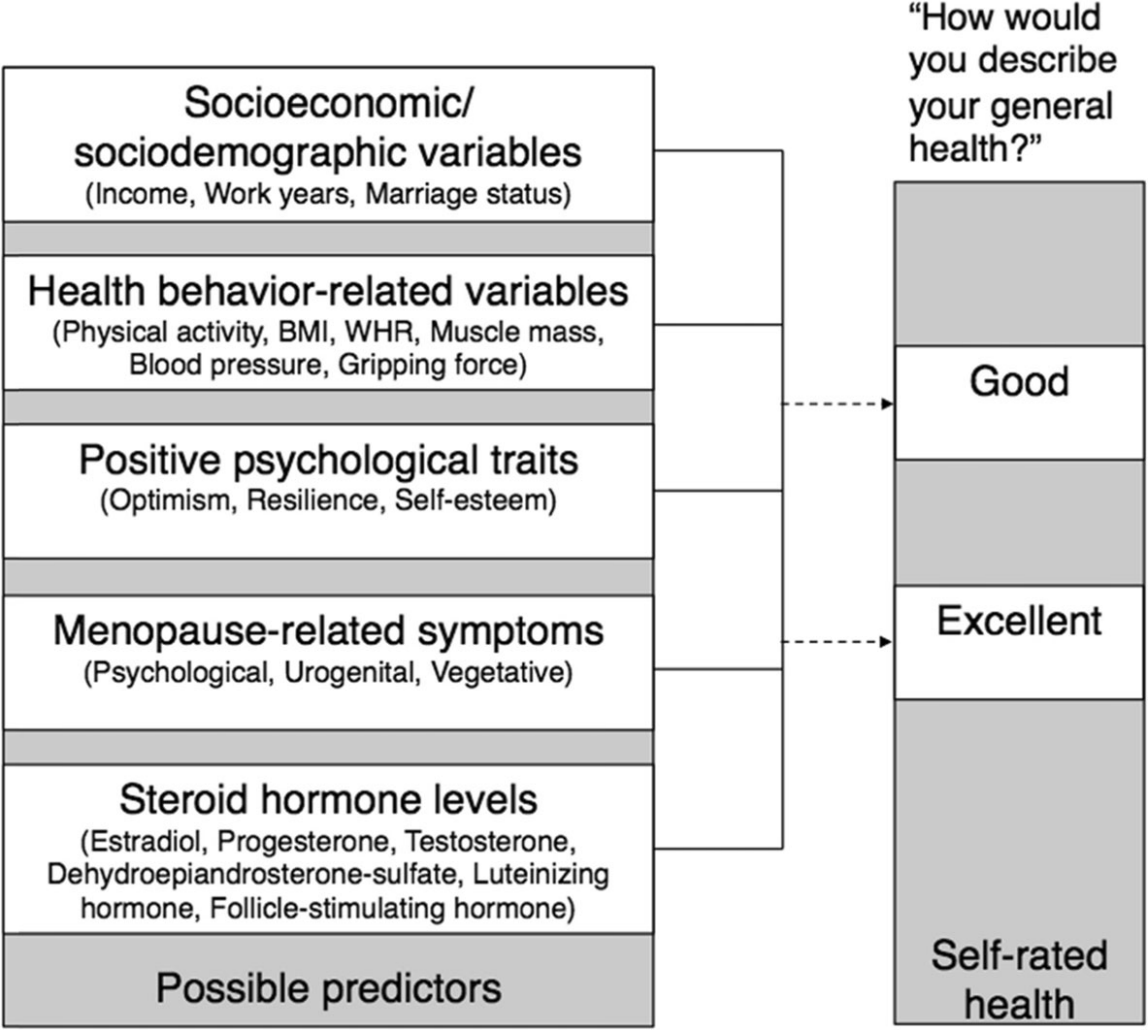
Pool of possible predictors of healthy aging as operationalized by self-rated health

Healthy aging is a major public health priority and studies in women are crucial, as they grow older but seem to be more susceptible to disease than men. Previous studies suggest socioeconomic and sociodemographic variables, health behavior-related variables, positive psychological traits, as well as steroid hormone and gonadotropin levels as possible predictors of healthy aging. Previous studies have yielded limited consensus on the predictors of healthy aging, possibly due to the focus on single variables or the combination of only a small number of variables. Additionally, most studies considered psychosocial and biological variables separately. The goal of this study is therefore to simultaneously consider biological and psychosocial variables, which have been suggested in the literature as possible predictors of healthy aging. We added menopausal symptoms, as they were never considered before as healthy aging predictors. Moreover, we also investigate whether the set of suitable predictors differs if women’s age and menopausal stage are taken into account (see Fig. 1).

## Methods

### The current study

This study is part of a larger project conducted at the University of Zurich [43]. The Women 40+ Healthy Aging Study investigated a subjectively healthy population of middle-aged and older women using a biopsychosocial approach. A cross-sectional research design was employed. Subjects were community-dwelling women aged between 40 and 75 years.

Participants were recruited via flyers, facebook, articles in health-related online portals and newsletters, as well as mailing lists. Participants were included in the study if they reported being free of any acute or chronic somatic disease or mental disorder and if they had not received any psychotherapeutic or psychopharmacological treatment during the last 6 months. The following exclusion criteria were applied: habitual alcohol intake of more than two standard units of alcohol per day, pregnancy in the last 6 months, precocious menopause, and a menopausal status due to surgical removal of either both ovaries or the uterus. Additionally, women were not eligible for the study if they had used either oral contraceptives or hormone therapy in the last 6 months. Prospective participants completed an online self-screening for the described criteria and were only included in the study if they met all criteria described above.

### Study procedure

After enrolment, a member of the study team conducted an additional telephone screening to minimize the risk of including women who were not suitable and to clarify open questions about the study procedure. Participants were subsequently invited to a laboratory session at the University of Zurich. All sessions started at 7:45 a.m. and followed a standardized protocol. Saliva and blood spot samples were collected at 8:00 a.m. to control for diurnal rhythm. Subsequently, a member of the study team performed the additional physiological assessments (described below). Moreover, participants were asked to complete an online survey encompassing validated psychological questionnaires in the days following the laboratory session. For premenopausal women, the laboratory session and the psychological assessments were conducted in the follicular phase of the menstrual cycle. The criteria which were applied to characterize the menopausal stages are described elsewhere [43].

The G*Power 3.1 software [44] was used to perform sample size calculations. Calculations were based on F-tests using linear multiple regression analysis with a fixed model and investigating an R^2^ increase. A total of 130 participants were recruited, of which nine had to be excluded from the analyses due to the intake of medication influencing the endocrine system. Therefore, a total of 121 participants were confirmed eligible and included in the present analyses, which was in line with previous sample size recommendations [45, 46]. Measurements were conducted between June 2017 and February 2018.

### Healthy aging measure

Healthy aging was operationalized via self-rated health. As shown by our workgroup and others, self-rated health is considered as a valid health indicator in middle-aged and older men and women [47–49]. We assessed self-rated health with the Short-Form Health Survey 36 (SF-36) item: “How would you describe your general health?”, with the response options “poor”, “fair”, “good”, “very good” or “excellent”. Only women with at least good self-rated health were included in our study. For the analyses, we distinguished participants with “very good” or “excellent” self-rated health (combined into one group and termed as excellent, *N* = 87) from women with “good” self-rated health (*N* = 34). Group membership was considered as dependent variable in the subsequent analyses. We chose this approach to gain a nuanced view of the particularities at the upper end of the illness-wellness continuum.

### Predictor variables

#### Socioeconomic and sociodemographic variables

In the online survey, participants were asked about their age, their personal annual income, and their annual household income (personal and partner’s) as indicators of socioeconomic status (SES). Moreover, we recorded the number of years the participants have worked so far. Participants were additionally asked about their marital status; they were considered as non-married for the analyses if they were “non-married”, “divorced”, or “widowed”, in line with Prus [50].

#### Health behavior-related variables

Participants also reported on the online survey how many hours per week they usually spend engaging in mild physical activity (such as walking or stretching), moderate physical activity (such as housework, Nordic walking, or cycling), or intense physical activity (such as tennis, running, or soccer). The additional health behavior-related variables were assessed during the laboratory session. BMI was calculated as kg/m^2^ and waist-to-hip ratio (WHR) was calculated as the circumference of the waist divided by the circumference of the hips. To complement BMI and WHR, we also assessed percentage of body fat and percentage of muscle mass using bioelectrical impedance analysis (BIA; Biacorpus RX 4000). This non-invasive approach allows body composition to be measured according to the distinct conductivity of different body compartments (i.e. fat and water). Blood pressure (systolic and diastolic; in mmHg) and pulse (in beats per minute, bpm) were assessed after at least 10 min of rest in a supine position (Medisana MTX). Grip strength was measured using a hydraulic hand dynamometer (LITE) to measure the strength (in kg) in the dominant arm in two consecutive attempts. The maximum grip strength was considered in the analyses.

#### Steroid hormones and gonadotropins

Saliva samples were used to analyze levels of estradiol (E2, pmol/L), progesterone (P4, pmol/L), cortisol (C, nmol/L), testosterone (T, pmol/L), and dehydroepiandrosterone sulfate (DHEAS, ng/ml). Samples were collected in 2-ml SaliCaps (IBL International GmbH, Hamburg, Germany) using the passive drool method and were subsequently stored at − 20 °C until biochemical analyses were performed. Thawed saliva samples were centrifuged and analyzed using immunoassays (IBL International GmbH, Hamburg, Germany). All salivary analyses were performed at our Biochemical Laboratory, Institute of Psychology, University of Zurich. Intra- and inter-assay variation was less than 10% and sensitivity was 1.10 pmol/L for E2, 8.24 pmol/L for P4, 0.03 nmol/L for C, 6.25 pmol/L for T, and 0.05 ng/mL for DHEAS. Dried blood spot (DBS) samples were assessed using a capillary blood sample to analyze the gonadotropins luteinizing hormone (LH, mlU/ml) and follicle-stimulating hormone (FSH, mlU/ml). A disposable lancet (Accu-Chek® Safe-T-Pro Plus) was used to draw a small blood sample from the participant’s finger, which was collected onto a standardized filter paper (Whatman® Protein Saver Cards, No. 903). Blood samples were then dried at room temperature for 4 hours. DBS samples were stored at a temperature of − 20 °C until biochemical analyses were performed at the biochemical laboratory Cytolab in Regensdorf, Switzerland. For comparability with other studies, LH and FSH values were converted to plasma equivalents according to the formula by Worthman and Stallings [51]:

$$ {\mathrm{LH}}_{\mathrm{plasma}}=0.07+1.90\ {\mathrm{LH}}_{\mathrm{DBS}}\ \mathrm{and}\ {\mathrm{FSH}}_{\mathrm{plasma}}=0.424+2.207\ {\mathrm{FSH}}_{\mathrm{DBS}}. $$

#### Positive psychological traits

The online survey included validated questionnaires on psychosocial factors. Dispositional optimism was assessed with the revised version of the 10-item Life Orientation Test (LOT-R [52]). Items are rated on a 5-point scale ranging from 1) *absolutely not applicable* to 5) *absolutely applicable*. We used the optimism subscale of the LOT-R in our analyses. Resilience was measured with the 11-item Resilience Scale (RS-11 [53]), with items rated on a 7-point scale from 1) *I don’t agree* to 7) *I absolutely agree*. The RS-11 is recommended for elderly populations [54]. Self-worth was measured with the Multidimensional Self-Esteem Scale (MSES [55]), which is a German translation and slight adaptation of the Multidimensional Self-Concept Scale [56]. The 32-item MSES assesses six different aspects of self-esteem: emotional self-esteem, social skills, social confidence, achievement-related self-esteem, physical attractiveness, and sportiness. Items are rated on a 7-point scale from 1) *not at all* to 7) *very*. The total score on the general self-esteem was used in this study.

#### Menopause-related symptoms

Menopause-related symptoms were assessed using the Menopause Rating Scale (MRS [57]). The MRS examines psychological, vegetative, and urogenital symptoms, with items rated on a 5-point scale from 0) *none* to 4) *very severe* symptoms. The sum score was considered for the present analyses. A sum score of 0 represents the minimum score and indicates that the woman is asymptomatic, while a value of 44 indicates the highest possible degree of symptoms.

#### Statistical analysis

Participants’ self-ratings of health represented the outcome variable of our study. Excellent and very good self-rated health were collapsed into one category, as initial logistic regression analyses indicated that the maximum likelihood estimates were almost identical for the top two categories when compared with the good self-rated health category. Therefore, membership in either the good self-rated health group or the excellent self-rated health group (comprising excellent and very good) was the dependent variable in the analyses. The good self-rated health group was considered as the reference group.

The main goal of the analyses was to reduce the pre-established set of possible predictors in order to find the simplest and most accurate model (principle of parsimony) to predict whether participants rated their health as excellent compared to good. To achieve this, we first examined the physiological values and excluded implausibly high physiological values from further analyses. To judge the endocrine values, the respective menopausal stage of the women was considered. This approach resulted in the exclusion of eight cases due to high values for DHEAS (*N* = 2), E2 (*N* = 2), and C (*N* = 2). Missing data was excluded listwise from subsequent analyses. Second, we performed correlation analyses between conceptually similar possible predictor variables. If two values were strongly correlated (*r* > .6), one was excluded. For decisions regarding retention or exclusion, we consulted the literature.

In a next step, possible predictors were z-transformed to enable comparison of the *b* values. To reduce the final set of possible predictors, a binary logistic regression using the forward conditional method was performed [58]. The regression analysis was performed three times, first using only the expected predictors, second adding *age* as a control variable, and finally adding *menopausal status* instead of age as a control variable. Menopausal stage was dummy-coded, with premenopause (compared to peri- and postmenopause) and postmenopause (compared to pre- and perimenopause) as dummies. The results of the regression models are presented as odds ratio Exp(B) and represent the exponential of the *b* for the predictor (*e*^*b*^). Odds ratios > 1 indicate that a one-unit increase in the predictor *increases* the odds of rating one’s health as excellent. Conversely, odds ratios < 1 indicate that a one-unit increase in the predictor *decreases* the odds of rating one’s health as excellent. In all analyses, the significance level was set at *p* < .05. Values of *p* < .10 were considered as trends. Analyses were performed using SPSS (Version 23, IBL).

## Results

### Demographic characteristics

Overall, the participants’ mean age was 53.23 years (range: 40–73). Fifty-four women were premenopausal, 10 were perimenopausal, and 57 were postmenopausal. Women with excellent self-rated health had a mean age of 53.67 (8.83) and women with good self-rated health had a mean age of 52.12 (9.39) years. This age difference was not statistically significant (*p* = .396). The list of possible predictors of self-rated health is depicted in Table 1 as means and standard deviations for the two groups of self-rated health separately.

**Table 1:** Set of possible predictors of self-rated health

Variable	Good	Excellent
Socioeconomic/ sociodemographic variables		
Income (CHF)	52,890.03 (33,106.61)	69,848.78 (40,669.19)
Income household (CHF)	98,781.56 (59,444.28)	139,475.51 (77,495.95)
Married (N)	15 (44.1%)	49 (56.3%)
Work years	29.21 (10.50)	29.11 (8.69)
Health behavior-related variables		
Activity intense (h/week)	1.61 (1.63)	3.06 (2.66)
Activity moderate (h/week)	8.16 (7.54)	6.76 (7.04)
Activity light (h/week)	5.03 (3.85)	5.52 (6.64)
BMI (kg/m^2^)	24.58 (4.76)	22.40 (2.98)
WHR (waist/hip)	.82 (.06)	.83 (.15)
Body fat (%)	31.56 (7.81)	27.71 (5.86)
Muscle mass (%)	49.41 (3.50)	49.67 (4.18)
Blood pressure systolic (mmHg)	120.15 (16.26)	119.80 (12.95)
Blood pressure diastolic (mmHg)	75.00 (9.71)	75.70 (9.03)
Pulse (bpm)	61.53 (8.56)	59.06 (8.79)
Grip strength (kg)	31.12 (5.82)	32.76 (4.90)
Steroid hormones and gonadotropins		
E2 (pmol/L)	6.96 (5.65)	5.98 (5.31)
P4 (pmol/L)	120.61 (125.77)	111.28 (113.35)
C (nmol/L)	7.03 (6.12)	7.03 (11.58)
T (pmol/L)	37.06 (32.04)	32.02 (26.07)
DHEAS (ng/ml)	1.94 (1.05)	1.34 (.84)
LH (mlU/ml)	.56 (.75)	.60 (.54)
FSH (mlU/ml)	3.08 (3.00)	4.08 (3.80)
Positive psychological traits		
Optimism	11.67 (1.99)	12.51 (1.84)
Resilience	61.03 (6.96)	65.77 (6.44)
Self-esteem	114.00 (16.30)	119.93 (17.60)
Menopausal symptoms		
Symptom total score	18.27 (5.45)	16.18 (4.26)

Note: Values refer to mean levels and standard deviations (SD) in participants with good (reference group) and excellent self-rated health. Abbreviations: Swiss francs (CHF) per year, Body mass index (BMI), Waist-to-hip ratio (WHR), Estradiol (E2), Progesterone (P4), Cortisol (C), Testosterone (T), Dehydroepiandrosterone sulfate (DHEAS), Luteinizing hormone (LH), Follicle-stimulating hormone (FSH). Total *N* = 121 except for E2, C, and DHEAS, where *N* = 119

### Selection of possible predictors

Steroid hormone and gonadotropin levels were all in the normal range to be expected for the age and menopausal stage of the participants. There were no statistically or clinically significant differences in endocrine markers between the two groups. Therefore, we did not expect to find any predictive value for the endocrine markers and decided not to include them in the subsequent analyses.

In a next step, we performed correlation analyses among sociodemographic variables, activity-related variables and positive psychological traits (see Table 2) in order to rule out potentially high intercorrelations. The correlation analysis among activity-related variables revealed a high correlation (*p* = .829) between BMI and percentage fat mass (see Table 2). BMI was subsequently retained for the analyses while percentage fat mass was excluded. All remaining variables were considered as possible predictors and were therefore included in the subsequent binary logistic regressions.

**Table 2:** Correlation analyses of health behavior-related variables

	1	2	3	4	5	6	7	8	9	10	11
Light activity (1)	1.00	.33^**^	.01	.07	.10	−.03	−.04	−.02	.010	.05	−.01
Moderate activity (2)		1.00	.20^*^	−.04	.06	−.21^*^	−.19^*^	−.09	−.08	−.06	.04
Intensive activity (3)			1.00	−.33^**^	−.10	−.10	−.11	−.38^**^	.04	−.44^**^	.18^*^
BMI (4)				1.00	.32^**^	.30^**^	.26^**^	.29^**^	−.03	.83^**^	.30^**^
WHR (5)					1.00	.19^*^	.15	.10	−.15	.33^**^	.04
Systolic blood pressure (6)						1.00	.81^**^	.22^**^	−.13	.27^**^	.07
Diastolic blood pressure (7)							1.00	.26^**^	.04	.23^**^	.05
Pulse (8)								1.00	−.05	.44^**^	−.07
Grip strength (9)									1.00	−.16^*^	.16^*^
Percentage fat mass (10)										1.00	−.12
Percentage muscle mass (11)											1.00

**. Correlation significant at .01 (one-tailed)

*. Correlation significant at .05 (one-tailed)

### Binary logistic regressions

A first binary logistic regression using the forward conditional method resulted in a model including BMI, intensive physical activity, and resilience as predictors (see Table 3, upper section). Using this model, 79.6% of the participants could be correctly classified as having either excellent or good self-rated health. The full model containing the three predictors was statistically significant (*χ*^*2*^(3) = 27.407, *R*^*2*^ = .37 (Nagelkerke), *R*^*2*^ = .26 (Cox & Snell), *p* < .001), indicating that the model was able to distinguish between participants with excellent and good self-rated health.

**Table 3:** Binary logistic regressions

Self-rated health	*b*	*p*	Exp(B)	95% CI for OR	
				Lower	Upper
Included					
Constant	1.29	<.001	3.62		
BMI	−.54	.06	.59	.33	1.03
Intensive activity	.82	.04	2.27	1.06	4.86
Resilience	.86	<.01	2.37	1.34	4.18
Included					
Constant	−1.68	.350	.19	.19	
Age	.51	.10	1.66	.91	3.06
BMI	−.60	.05	.55	.30	1.01
Intensive activity	1.01	.02	2.75	1.19	6.34
Resilience	.80	<.01	2.23	1.24	4.01
Included					
Constant	1.44	.12	4.24		
Premenopausal	−.66	.51	.52	.07	3.69
Postmenopausal	.43	.67	1.54	.21	11.28
BMI	−.60	.05	.55	.30	.10
Intensive activity	.98	.02	2.66	1.17	6.04
Resilience	.88	<.01	2.40	1.33	4.33

Note: Results of binary logistic regression without control variables (upper section) and with age (middle section) and menopausal stage (lower section) as control variables

In a second binary logistic regression, age was included as a control variable when the forward conditional method was applied. The regression analysis revealed a model with BMI, intensive physical activity, and resilience as predictors (see Table 3, middle section). Using this age-controlled model, 77.4% of the participants could be correctly classified as having excellent or good self-rated health. The full model containing the three predictors and *age* was statistically significant (*χ*^*2*^(4) = 30.288, *R*^*2*^ = .40 (Nagelkerke), *R*^*2*^ = .28 (Cox & Snell), *p* < .001), indicating that this model was able to distinguish between women with excellent and good self-rated health.

In a third binary logistic regression, the dummy-coded menopausal status was included as a control variable when applying the forward conditional method. The regression revealed a model with BMI, intensive physical activity, and resilience as predictors (see Table 3, lower section). With this model controlling for menopausal status, 77.4% of the participants could be correctly classified as having excellent or good self-rated health. The full model containing the three predictors and *menopausal status* was statistically significant (*χ*^*2*^(5) = 30.59, *R*^*2*^ = .40 (Nagelkerke), *R*^*2*^ = .28 (Cox & Snell), *p* < .001), indicating that the model was able to distinguish between participants with excellent and good self-rated health.

## Discussion

The aim of the present analyses was to simultaneously consider biological and psychological variables in order to predict self-rated health at the top end of the illness-wellness continuum. BMI, intensive physical activity, and resilience were distinguishing factors between the two groups of either excellent or good self-rated health. There were no clinically significant differences between women with excellent and good self-rated health regarding steroid hormone and gonadotropin levels.

Our findings on physical activity and BMI are in line with previous research. Regular physical exercise has been shown to have positive effects in terms of the prevention of depression [59] and of cognitive decline [60, 61], hormonal balance [62], the prolongation of the life span [63], and increased odds of being a healthy ager [64]. Moreover, reduced calorie intake [65] and a balanced and nutrient-rich diet [66, 67] seem to have positive effects on health in older age. These health-promoting effects are possibly mediated by positive effects of exercise and a lower BMI on skeletal muscle [68], bone health [69], as well as a more balanced psychological and physiological stress response [61].

Positive psychological traits are important factors for maintaining health throughout the aging process. Previous studies already demonstrated the importance of positive psychological traits such as self-efficacy, optimism [70], and positive affect [49] for future health in older age. Resilient individuals seem to redefine themselves more easily and remain more independent in older age by maintaining self-efficacy and self-esteem [71]. Resilience might be especially important in midlife, since this represents a period of pronounced changes in a woman’s life: The loss of reproductive function with menopause is approaching and children may be beginning to leave home, to name just two possible stressors. Each woman will perceive these changes differently, as either relieving or distressing. Irrespective of valence, however, resilient women might cope better with these life changes in general, by adapting to the new circumstances and therefore maintaining health and wellbeing [15, 34]. Interventions specifically targeting resilience in older age are lacking so far. In this respect, it is important to refer to the publication of MacLeod and collegues, who proposed a framework to guide resilience interventions specifically in older adults [72].

Interestingly, endocrine parameters were not predictive of the subjective health status. The present study is one of the first to explicitly consider steroids and gonadotropins as markers of healthy aging in women. Despite the value of steroids and gonadotropins in predicting pathological states such as depression [73, 74] or cognitive decline [30, 31], these markers do not seem to have predictive ability for generally healthy women. If the endocrine parameters are in the normal range expected for the age and menopausal stage, as was the case for this sample, other factors appear to be of greater importance. Although endocrine levels change considerably with age and menopause, not all women are negatively affected by these endocrine changes. While most studies on endocrine changes in midlife women have focused on vulnerable subjects who are at risk of developing pathologies, our findings highlight that women can remain healthy despite hormonal changes. Longitudinal analyses of our cohort are planned, which will provide insights into how healthy endocrine aging might look.

There are various potential explanations for the lack of associations with the remaining predictors of self-rated health investigated in the present study. The participants of this study were relatively homogenous in many aspects: The majority were in an intimate relationship and had a household income above the mean level for Switzerland (around 84,000 CHF/ 84,739 $). Although some menopause-related symptoms were reported, the average symptom score was very low in our sample and only a small number of participants reported bothersome menopausal complaints. To the best of our knowledge, this is the first study to consider menopause-related symptoms as predictors of self-rated health. Given the frequency of bothersome menopausal complaints in the general population (see i.e. [24–27]), future studies on predictors of healthy aging should nonetheless take menopause-related symptoms into account, especially when examining more diverse samples with regard to health status.

### Strengths and limitations

One major strength of the present study lies in its focus on healthy aging. By investigating a generally healthy population of midlife women, we were able to draw conclusions regarding physiological, psychological, and behavioral factors distinguishing between women with excellent self-rated health and women with good self-related health. This highlights the notion of healthy aging to go beyond the avoidance of disability and disease. The sample comprised a large age range and included pre-, peri-, and postmenopausal women, thus allowing us to disentangle the effects of chronological and endocrine age on subjective health. The strict inclusion criteria helped to keep the noise from interventions and treatments for subjective health to a minimum. Moreover, positive criteria such as positive psychological traits were included in order to identify high functioning at the top end of the health spectrum.

The reported findings result from cross-sectional analyses. Although the sample size was sufficiently powered for the analyses, the distribution of participants between the two groups of self-rated can be discussed as possible limitation. The planned follow-up of the same sample will clarify whether our established set of variables have predictive power for future health outcomes and individual health trajectories over time. Moreover, more frequent sampling of the endocrine measures could help to clarify whether generally healthy midlife women have a distinct endocrine profile. Based on previous research (i.e. [28]), it might be the case that rather stable levels of steroids and gonadotropins constitute a unifying characteristic.

## Conclusion

Healthy aging has become a major public health priority due to the prolongation of the life span and today’s larger elderly population compared to previous generations. The prolongation of the healthy life span in women should be a major public health focus. In the present study, BMI, intensive physical activity, and resilience emerged as distinguishing factors between women with excellent and good self-rated health. BMI and regular physical activity can be actively targeted by lifestyle modifications, and our findings suggest that the development of resilience training for older adults would be beneficial. Although endocrine levels change considerably with age and menopause, not all women are negatively affected by these endocrine changes. Studies in healthy aging women are still scarce. Such studies are important, as they can foster our knowledge on contributors of a prolonged health span. Future healthy aging studies should incorporate the physiological, psychosocial, and behavioral levels, thereby taking a more inclusive approach, which acknowledges the multiple facets of health in midlife and older age.

## Data Availability

The datasets used and/or analyzed during the current study are available from the corresponding author on reasonable request.

## Abbreviations

BMI: Body Mass Index
bpm: Beats per minute
C: Cortisol
DBS: Dried Blood Spots
DHEAS: Dehydroepiandrosterone sulfate
E2: Estradiol
FSH: Follicle-stimulating hormone
HPG: Hypothalamic-Pituitary-Gonadal axis
LH: Luteinizing hormone
LOT-R: Life Orientation Test Revised
MRS: Menopause Rating Scale
MSCS: Multidimensional Self-Concept Scale
MSES: Multidimensional Self-Esteem Scale
P4: Progesterone
RS-11: Resilience Scale
SF-36: Short-Form Health Survey 36
T: Testosterone
WHO: World Health Organization
WHR: Waist-to-hip ratio

## References

[1] Beard JR, Officer A, De Carvalho IA, Sadana R, Pot AM, Michel JP, Lloyd-Sherlock P, Epping-Jordan JE, Peeters GMEE, Mahanani WR, Thiyagarajan JA, Chatterji S (2016). The world report on ageing and health: a policy framework for healthy ageing. Lancet..

[2] Beard JR, Officer AM, Cassels AK (2016). The world report on ageing and health. Gerontologist..

[3] Engel GL (1981). The clinical application of the biopsychosocial model. J Med Philos (United Kingdom).

[4] Rowe JW, Kahn RL (1987). Human aging: Usual and successful. Science (80- ).

[5] Kalache A, Gatti A (2003). Active ageing: a policy framework. Adv Gerontol.

[6] Van Oyen H, Nusselder W, Jagger C, Kolip P, Cambois E, Robine JM (2013). Gender differences in healthy life years within the EU: an exploration of the “health-survival” paradox. Int J Public Health.

[7] Austad SN (2006). Why women live longer than men: sex differences in longevity. Gend Med.

[8] Jylhä M (2009). What is self-rated health and why does it predict mortality? Towards a unified conceptual model. Soc Sci Med.

[9] DeSalvo KB, Bloser N, Reynolds K, He J, Muntner P (2006). Mortality prediction with a single general self-rated health question: a meta-analysis. J Gen Intern Med.

[10] Idler EL, Benyamini Y (1997). Self-rated health and mortality: a review of twenty-seven community studies. J Health Soc Behav.

[11] Pinquart M (2001). Correlates of subjective health in older adults: a meta-analysis. Psychol Aging.

[12] Molarius A, Janson S (2002). Self-rated health, chronic diseases, and symptoms among middle-aged and elderly men and women. J Clin Epidemiol.

[13] Barsky AJ, Cleary PD, Klerman GL (1992). Determinants of perceived health status of medical outpatients. Soc Sci Med.

[14] Bryant LL, Beck A, Fairclough DL (2000). Factors that contribute to positive perceived health in an older population. J Aging Health.

[15] Jeste DV, Savla GN, Thompson WK, Vahia IV, Glorioso DK, Martin AS, Palmer BW, Rock D, Golshan S, Kraemer HC, Depp CA (2013). Association between older age and more successful aging: critical role of resilience and depression. Am J Psychiatry.

[16] Depp CA, Jeste DV (2006). Definitions and predictors of successful aging: a comprehensive review of larger literature. Am J Geriatr Psychiatry.

[17] Depp CA, Glatt SJ, Jeste DV (2007). Recent advances in research on successful or heatlhy aging. Curr Psychiatry Rep.

[18] Burke GL, Arnold AM, Bild DE, Cushman M, Fried LP, Newman A, Nunn C, Robbins J (2001). Factors associated with healthy aging: the cardiovascular health study. J Am Geriatr Soc.

[19] Beyer AK, Wolff JK, Warner LM, Schüz B, Wurm S (2015). The role of physical activity in the relationship between self-perceptions of ageing and self-rated health in older adults. Psychol Health.

[20] Rubinow DR, Schmidt PJ (2006). Gonadal steroid regulation of mood: the lessons of premenstrual syndrome. Front Neuroendocrinol.

[21] Schmidt PJ, Ben Dor R, Martinez PE, Guerrieri GM, Harsh VL, Thompson K, Koziol DE, Nieman LK, Rubinow DR (2015). Effects of estradiol withdrawal on mood in women with past perimenopausal depression: a randomized clinical trial. JAMA Psychiatry.

[22] Soares C, Zitek B (2008). Reproductive hormone sensitivity and risk for depression across the female life cycle: a continuum of vulnerability?. J Psychiatry Neurosci.

[23] Hoyt LT, Falconi AM (2015). Puberty and perimenopause: reproductive transitions and their implications for women’s health. Soc Sci Med.

[24] Lupien SJ, Nair NP, Brière S, Maheu F, Tu MT, Lemay M, McEwen BS, Meaney MJ (1999). Increased cortisol levels and impaired cognition in human aging: implication for depression and dementia in later life. Rev Neurosci.

[25] Sarri G, Pedder H, Dias S, Guo Y, Lumsden MA (2017). Vasomotor symptoms resulting from natural menopause: a systematic review and network meta-analysis of treatment effects from the National Institute for health and care excellence guideline on menopause. BJOG An Int J Obstet Gynaecol.

[26] Schmidt PJ, Rubinow DR (2009). Sex hormones and mood in the perimenopause. Ann N Y Acad Sci.

[27] Soares CN (2017). Depression and menopause: current knowledge and clinical recommendations for a critical window. Psychiatr Clin North Am.

[28] Gordon JL, Rubinow DR, Eisenlohr-Moul TA, Leserman J, Girdler SS (2016). Estradiol variability, stressful life events, and the emergence of depressive symptomatology during the menopausal transition. Menopause..

[29] Bromberger JT, Schott LL, Kravits HM, Sowers M, Avis NE, Gold EB, Randolph JF, Matthews KA (2010). Longitudinal change in reproductive hormones and depressive symptoms across the menopausal transition. Arch Gen Psychiatry.

[30] Hu J, Chu K, Song Y, Chatooah ND, Ying Q, Zhou J, Qu F, Zhou J, Hu J, Chu K, Song Y, Chatooah ND, Ying Q, Ma L, Zhou J, Qu F (2017). Higher level of circulating estradiol is associated with lower frequency of cognitive impairment in Southeast China. Gynecol Endocrinol.

[31] Henderson VW, St. John JA, Hodis HN, McCleary CA, Stanczyk FZ, Karim R, Shoupe D, Kono N, Dustin L, Allayee H, Mack WJ (2013). Cognition, mood, and physiological concentrations of sex hormones in the early and late postmenopause. Proc Natl Acad Sci.

[32] Fiacco S, Walther A, Ehlert U (2018). Steroid secretion in healthy aging. Psychoneuroendocrinology..

[33] Solberg Nes L, Segerstrom SC (2006). Dispositional optimism and coping: a meta-analytic review. Personal Soc Psychol Rev.

[34] Bonanno GA (2004). Loss, trauma, and human resilience: have we underestimated the human capacity to thrive after extremely adverse events?. Am Psychol.

[35] Scheier MF, Carver CS (1985). Optimism, coping, and health: assessment and implications of generalized outcome expectancies. Health Psychol.

[36] Rasmussen HN, Scheier MF, Greenhouse JB (2009). Optimism and physical health: a meta-analytic review. Ann Behav Med.

[37] Baumeister RF, Tice DM, Hutton DG (1989). Self-presentational motivations and personality differences in self-esteem. J Pers.

[38] Orth U, Robins RW, Widaman KF (2012). Life-span development of self-esteem and its effects on important life outcomes. J Pers Soc Psychol.

[39] Mäkikangas A, Kinnunen U, Feldt T (2004). Self-esteem, dispositional optimism, and health: evidence from cross-lagged data on employees. J Res Pers.

[40] Lu H, Li X, Wang Y, Song Y, Liu J (2018). The hippocampus underlies the association between self-esteem and physical health. Sci Rep.

[41] Orth U, Robins RW, Meier LL, Conger RD (2016). Refining the vulnerability model of low self-esteem and depression: disentangling the effects of genuine self-esteem and narcissism. J Pers Soc Psychol.

[42] Karatas Z, Tagay O (2012). Self esteem, locus of control and multidimensional perfectionism as the predictors of subjective well being. Int Educ Stud.

[43] Mernone L, Fiacco S, Ehlert U (2019). Psychobiological factors of sexual health in aging women - findings from the women 40+ healthy aging study. Front Psychol.

[44] Faul F, Erdfelder E, Buchner A, Lang A-GA (2009). G*Power. Behav Res Methods.

[45] Vittinghoff E, McCulloch CE (2007). Relaxing the rule of ten events per variable in logistic and cox regression. Am J Epidemiol.

[46] van Smeden M, Moons KGM, de Groot JAH, Collins GS, Altman DG, Eijkemans MJC, Reitsma JB (2018). Sample size for binary logistic prediction models: beyond events per variable criteria. Stat Methods Med Res.

[47] Idler EL, Russell LB, Davis D (2000). Survival, functional limitations, and self-rated health in the NHANES I epidemiologic follow-up study, 1992. Am J Epidemiol.

[48] Miilunpalo S, Vuori I, Oja P, Pasanen M, Urponen H (1997). Self-rated health status as a health measure: the predictive value of self-reported health status on the use of physician services and on mortality in the working-age population. J Clin Epidemiol.

[49] Dainese SM, Allemand M, Ribeiro N, Bayram S, Martin M, Ehlert U (2011). Protective factors in midlife. GeroPsych (Bern).

[50] Prus SG (2011). Comparing social determinants of self-rated health across the United States and Canada. Soc Sci Med.

[51] Worthman CM, Stallings JF (1994). Measurement of gonadotropins in dried blood spots. Clin Chem.

[52] Glaesmer H, Hoyer J, Klotsche J, Herzberg PY (2008). Die deutsche Version des Life-Orientation-Tests (LOT-R) zum dispositionellen Optimismus und Pessimismus. Zeitschrift fur Gesundheitspsychologie.

[53] Wagnild G, Young H (1993). Development and psychometric evaluation of RS scale. J Nurs Meas.

[54] Cosco TD, Kaushal A, Richards M, Kuh D, Stafford M (2016). Health Qual Life Outcomes.

[55] Schütz A, Sellin I (2006). Multidimensionale Selbstwertskala. Z Klin Psychol Psychother.

[56] Fleming JS, Courtney BE (1984). The dimensionality of self-esteem: II. Hierarchical facet model for revised measurement scales. J Pers Soc Psychol.

[57] Schneider HP, Heinemann LA, Rosemeier HP, Potthoff P, Behre HM (2000). The menopause rating scale (MRS): reliability of scores of menopausal complaints. Climacteric..

[58] Hosmer DW, Lemeshow S, Sturdivant RX (2013). Applied logistic regression.

[59] Mammen G, Faulkner G (2013). Physical activity and the prevention of depression. Am J Prev Med.

[60] Dumas JA (2017). Strategies for preventing cognitive decline in healthy older adults. Can J Psychiatr.

[61] Kennedy G, Hardman RJ, MacPherson H, Scholey AB, Pipingas A (2016). How does exercise reduce the rate of age-associated cognitive decline? A review of potential mechanisms. J Alzheimers Dis.

[62] Walther A, Lacker TJ, Ehlert U (2018). Everybody was kung-Fu fighting - the beneficial effects of tai chi qigong and self-defense kung-Fu training on psychological and endocrine health in middle aged and older men. Complement Ther Med.

[63] Gu D, Zhang Z, Zeng Y (2009). Access to healthcare services makes a difference in healthy longevity among older Chinese adults. Soc Sci Med.

[64] Daskalopoulou C, Stubbs B, Kralj C, Koukounari A, Prince M, Prina AM (2017). Physical activity and healthy ageing: a systematic review and meta-analysis of longitudinal cohort studies. Ageing Res Rev.

[65] Fontana L, Partridge L (2015). Promoting health and longevity through diet: from model organisms to humans. Cell..

[66] Lehert P, Villaseca P, Hogervorst E, Maki P, Henderson VW (2016). Individually modifiable risk factors to ameliorate cognitive aging: a systematic review and meta-analysis. Climacteric..

[67] Mujcic R, Oswald AJ (2019). Does eating fruit and vegetables also reduce the longitudinal risk of depression and anxiety? A commentary on “Lettuce be happy.”. Soc Sci Med.

[68] Cartee GD, Hepple RT, Bamman MM, Zierath JR (2016). Exercise promotes healthy aging of skeletal muscle. Cell Metab.

[69] Shapses SA, Pop LC, Wang Y (2017). Obesity is a concern for bone health with aging. Nutr Res.

[70] Bowling Ann, Iliffe Steve (2011). Psychological approach to successful ageing predicts future quality of life in older adults. Health and Quality of Life Outcomes.

[71] Becker G (1997). Disrupted lives: how people create meaning in a chaotic world.

[72] MacLeod S, Musich S, Hawkins K, Alsgaard K, Wicker ER (2016). The impact of resilience among older adults. Geriatr Nurs (Minneap).

[73] Avis NE, Crawford S, Stellato R, Longcope C (2001). Longitudinal study of hormone levels and depression among women transitioning through menopause. Climacteric..

[74] Freeman E, Sammel M, Lin H, Nelson D (2006). Associations of hormones and menopausal status with depressed mood in women with no history of depression. Arch Gen Psychiatry.

